# Factors related to hypermetabolism in individuals with type 2 diabetes mellitus and non-alcoholic fatty liver disease

**DOI:** 10.1038/s41598-023-30945-w

**Published:** 2023-03-04

**Authors:** Asieh Mansour, Soudabe Motamed, Azita Hekmatdoost, Sara Karimi, Mohammad Reza Mohajeri-Tehrani, Mohammad Abdollahi, Reihane Jelodar, Sayed Mahmoud Sajjadi-Jazi

**Affiliations:** 1 Endocrinology and Metabolism Research Center, Endocrinology and Metabolism Clinical Sciences Institute, Tehran University of Medical Sciences, Tehran, Iran; 2 Asadabad School of Medical Sciences, Asadabad, Iran; 3 Department of Clinical Nutrition & Dietetics, Faculty of Nutrition Sciences and Food Technology, National Nutrition and Food Technology Research Institute, Shahid Beheshti University of Medical Sciences, Tehran, Iran; 4 Digestive Disease Research Center, Digestive Disease Research Institute, Tehran University of Medical Sciences, Tehran, Iran; 5 Department of Internal Medicine, Shariati Hospital, Tehran University of Medical Sciences, Tehran, Iran

**Keywords:** Endocrinology, Gastroenterology, Medical research

## Abstract

Considering the progressive prevalence and co-occurrence of type 2 diabetes mellitus (T2DM) and non-alcoholic fatty liver disease (NAFLD), as well as the current evidence suggesting the elevated levels of basal metabolic rate (BMR) among these individuals, the present study aimed to identify factors determining hypermetabolism in such subjects. This cross sectional study was conducted in 30 to 53-year-old individuals with concurrent T2DM and NAFLD (controlled attenuation parameter score ≥ 260 dB/m). Resting energy expenditure (REE) was determined by an indirect calorimetry device. Hypermetabolism was defined as an elevated measured REE > 110% of the predicted REE. The multivariate logistic regression test was used for detecting factors associated with hypermetabolism. Between September, 2017, and March, 2018, a total of 95 eligible participants (64.40% male) with both T2DM and NAFLD were included, while 32.63% of them were classified as hypermetabolic. Overall, the mean recruitment age ± standard deviation and median (interquartile range) body mass index were 44.69 ± 5.47 years and 30.20 (27.80–33.30) kg/m^2^, respectively. Demographic, anthropometric and biochemical variables did not vary significantly across two groups except for total body water, low-density lipoprotein cholesterol and dipeptidyl peptidase 4 (DPP-4) inhibitors (p < 0.05). According to the results of multivariable logistic regression analyses, hypermetabolism had a positive association with adiponectin (odds ratio [OR] 1.167, 95% confidence interval [CI] 1.015–1.342, p = 0.030), physical activity (OR 1.134, 95% CI 1.002–1.284, p = 0.046), alanine transaminase (OR 1.062, 95% CI 1.006–1.122, p = 0.031) and diastolic blood pressure (OR 1.067, 95% CI 1.010–1.127, p = 0.021). However, fat free mass was inversely related to hypermetabolism (OR 0.935, 95% CI 0.883–0.991, p = 0.023). Adiponectin, alanine transaminase, physical activity, diastolic blood pressure and fat free mass were independently associated with hypermetabolism in subjects with NAFLD and T2DM.

## Introduction

Type 2 diabetes mellitus (T2DM) and non-alcoholic fatty liver disease (NAFLD) are both progressive health problems worldwide^1,2^. In addition, there is an intricate and reciprocal association between T2DM and NAFLD^3–5^. The prevalence of T2DM among NAFLD and non-alcoholic steatohepatitis (NASH) cases has been reported to be 22.5% and 43.6%, respectively^6^. The prevalence of NAFLD including liver steatosis and liver fibrosis in T2DM has been estimated to be more considerable^3,7^. According to the recent evidence, over fifty percent of individuals with T2DM concurrently have NAFLD^8,9^. NAFLD is substantially caused by visceral adipose tissue and insulin resistance^10^. It has been shown that individuals with both T2DM and NAFLD are more likely to experience progressive liver failure, liver fibrosis, cardiovascular diseases and diabetes complications, leading to the increased risk of morbidity and mortality^11–13^.

There is some evidence suggesting the increased basal metabolic rate (BMR) in subjects with T2DM^14,15^ and liver diseases^16,17^. BMR is defined as the amount of energy the body consumes at complete rest in a thermally neutral environment after 10–12 h of fasting and a night sleep^18^. BMR accounts for approximately 70% of total energy expenditure (TEE); it is considered as the main parameter determining appropriate energy requirement^17,19^. In general, some variables including age, sex, race, weight, height and body composition have been recognized as the most important predictive factors for BMR^19^. Today, in most studies, as well as in clinical practice, resting energy expenditure (REE), also known as resting metabolic rate (RMR), is used as an estimate of BMR, because it requires less restrictive conditions^18,20^.

BMR has been reported to increase by 5–7% in individuals with T2DM^21,22^. A significant correlation between BMR and homeostatic model assessment for insulin resistance (HOMA-IR), fasting blood sugar (FBS) and hemoglobin A1c (HbA1c) has been suggested^14^. Also, few studies have been designed to determine BMR in subjects with liver disease^17^. In a case–control study matched for sex, age, and body mass index (BMI), NAFLD cases had higher REE when compared to controls^16^.

Impaired glycemic control and elevated glucose output from the liver due to increased gluconeogenesis and glycogenolysis, which is caused by insulin inefficiency, as well as higher protein turnover, higher sympathetic nervous system activity, oxidative stress, visceral fat and altered body metabolism are potential mechanisms which have been suggested for the increased metabolism in diabetic subjects^21–23^. Other conditions including infection and inflammation, which are common in obese, diabetic and NAFLD individuals, are also suggested as possible factors that increase metabolism^16,17^.

In spite of the prevalence of T2DM and NAFLD and the importance of estimating appropriate energy expenditure, there is not much literature on exploring factors affecting metabolism in such diseases. Therefore, the objective of the present study was to find the predictive or determining factors that could affect hypermetabolism in subjects with both T2DM and NAFLD.

## Methods

This cross-sectional study was conducted in accordance with the Declaration of Helsinki and was approved by the Ethics Review Board at the Tehran University of Medical Sciences (IR.TUMS.MEDICINE.REC.1399.1281). Written informed consent was obtained from all participants.

Inclusion criteria included 30 to 53-year-old individuals with concurrent NAFLD, according to controlled attenuation parameter (CAP) score ≥ 260 dB/m^24^, and T2DM, based on American Diabetes Association criteria^25^. The exclusion criteria were as follows: being under insulin therapy, experiencing pregnancy or lactation, having the history of or currently drinking excessive alcohol (average drinking of alcohol over 20 g/day^26^), viral hepatitis infection, having the history of thyroid disease or limb amputation, suffering from cardiovascular diseases, kidney diseases, cancer, mental disorders, cirrhosis and other chronic liver diseases, and currently consuming supplements including vitamin E and other antioxidants.

### Clinical and laboratory data

The data about demography, past medical history and drug consumption were collected through face-to-face interviews. The height and weight of the participants were measured with the accuracy of 0.1 cm and 0.1 kg, respectively. BMI was calculated as weight in kg divided by the square of height in meters. Waist circumference (WC) was measured at the midpoint between the lower costal margin and the iliac crest. Body composition was estimated by a bioelectrical impedance scale (BC 418 MA, Tanita, Japan). To ensure hydration and obtain a valid evaluation, subjects were asked to follow the instructions before the body composition assessment as previously described^27^. The short version of the international physical activity questionnaire (IPAQ) was used to measure the physical activity of participants^28^. Systolic and diastolic blood pressure of the participants was measured on the left arm after at least 10 min of the seated rest. Current smoker was attributed to those who had smoked cigarette regularly in the past 6 months.

After overnight fasting, intravenous blood was drawn to measure enzymes and biomarkers. The enzyme-linked immunosorbent assay (ELISA) kit (Roche, Germany) was used to measure the serum concentration of liver enzymes, creatinine, high-sensitive C-reactive protein (hs-CRP) and lipids. A high performance liquid chromatography analyzer (Tosoh, Japan) was used to measure the levels of HbA1c. The FBS was measured using an autoanalyzer (Cobas c 311, Switzerland). The serum levels of insulin, C-peptide and thyroid-stimulating hormone (TSH) were determined by using ELISA kit (Monobind Inc., USA). Insulin resistance was estimated using the following formula: HOMA-IR = [Fasting insulin (μU/mL) × fasting glucose (mmol/L)/22.5].

### REE measurement

REE was measured by indirect calorimetry using a Fitmate calorimeter (Cosmed, Italy). After overnight fasting (at least 10 h), oxygen consumption and carbon dioxide production were obtained for 20 min with the participants lying down, without falling asleep, on a bed in a quiet environment. The first 5 min were excluded from the analysis. The Weir formula^29^ was then used to calculate REE. Predicted REE was calculated using the Harris–Benedict equations^30^. Hypermetabolism was defined as an elevated measured REE > 110% of predicted REE^31^.

### Transient elastography

Transient elastography was performed by one experienced operator, using FibroScan 502 instrument (EchoSense, France, 5 MHz), to detect and quantify liver steatosis (CAP, dB/m) and fibrosis (LSM, kPa).

### Data analysis

To check the normality of continuous variables, the Kolmogorov–Smirnov test was applied. The continuous variables with normal distribution were presented as mean ± standard deviation (SD) and those with non-normal distribution were presented as median (interquartile range [IQR]). The categorical variables were reported as number (%). Independent sample t-test, Mann–Whitney U test or Chi-square (χ^2^) test was used for comparison between groups as appropriate. Univariate logistic regression analysis was used to identify potential variables that have an effect on hypermetabolism. Variables with a p-value < 0.2 in univariate analysis were entered into the multivariate model using the backward selection approach. A p-value < 0.05 was considered statistically significant.

## Results

Between September, 2017, and March, 2018, a total of 108 subjects with both T2DM and NAFLD who met the inclusion criteria and were willingness to participate in the study were recruited from diabetes clinics in the Endocrinology and Metabolism Research Center, Tehran University of Medical Sciences (Tehran, Iran). Of these, 13 subjects were excluded from the study due to incomplete data and 95 participants were included in the final analysis. Demographic, anthropometric and biochemical characteristics of the participants with non-hypermetabolism (n = 64) and hypermetabolism (n = 31) are presented and compared in Table 1. The mean age of the participants was 44.69 ± 5.47 years, and 67.40% of them were males. The median BMI of the participants was 30.20 (27.80–33.30) kg/m^2^, while 52.63% of them were obese (BMI > 30 kg/m^2^). Among anthropometric measures, including height, weight, BMI, fat mass, fat percent, fat free mass (FFM), total body water (TBW) and WC, only TBW was significantly higher in subjects with non-hypermetabolism, as compared with hypermetabolism subjects (46.15 ± 8.09 vs. 42.09 ± 8.47, p = 0.027). In addition, among biochemical markers, subjects with non-hypermetabolism had a significantly higher level of low-density lipoprotein cholesterol (LDL-C), as compared with hypermetabolic cases (98.00 [68.00–118.00] vs. 71.00 [61.00–105.00], p = 0.041). The levels of FBS, fasting insulin, C-peptide, HOMA-IR, HbA1c, alanine transaminase (ALT), aspartate aminotransferase (AST), gamma-glutamyl transferase (GGT), total cholesterol, high-density lipoprotein cholesterol (HDL-C), triglycerides, creatinine, TSH, tumor necrosis factor alpha (TNF-α), adiponectin and hs-CRP were not significantly different between non-hypermetabolism and hypermetabolism groups (p > 0.05). In terms of drug consumption, there was a significant difference in the usage percentage of dipeptidyl peptidase 4 (DPP-4) inhibitors between the two groups (it was 51.6% in hypermetabolism subjects, while it was 15.6% in non-hypermetabolism subjects, p < 0.001).

**Table 1:** Demographic, anthropometric and biochemical characteristics of patients with NAFLD and diabetes, according to metabolism categories.

Characteristics	Total (n = 95)	Non-hypermetabolism (n = 64)	Hypermetabolism (n = 31)	p-value
Age (years)^a^	44.69 ± 5.47	44.06 ± 5.77	46.00 ± 4.60	0.106
Male (%)^b^	64 (67.4)	46 (71.9)	18 (58.1)	0.178
Smoking, current (%)^b^	25 (26.3)	16 (25)	9 (29)	0.676
Physical activity (METs h/day)^a^	31.49 ± 4.57	30.90 ± 4.54	32.71 ± 4.44	0.071
SBP (mmHg)^c^	120.00 (110.00–130.00)	120.00 (110.00–130.00)	120.00 (110.00–130.00)	0.965
DBP (mmHg)^a^	80.00 (80.00–90.00)	80.00 (76.25–90.00)	90.00 (80.00–95.00)	0.051
Height (cm)^c^	171.00 (165.00–177.00)	172.00 (167.25–178.00)	170.00 (161.00–176.00)	0.106
Weight (kg)^c^	88.00 (80.60–97.30)	89.75 (80.40–100.87)	86.70 (83.60–93.10)	0.300
BMI (kg/m^2^)^c^	30.20 (27.80–33.30)	29.70 (27.45–33.17)	31.20 (28.30–33.50)	0.552
Fat mass (kg)^a^	27.28 ± 8.37	26.90 ± 8.43	28.04 ± 8.33	0.540
Fat percent (%)^c^	28.55 (24.15–37.12)	28.40 (24.70–35.70)	29.00 (23.70–41.70)	0.279
FFM (kg)^a^	61.73 ± 10.78	63.16 ± 10.90	58.81 ± 10.07	0.066
TBW (kg)^a^	44.80 ± 8.40	46.15 ± 8.09	42.09 ± 8.47	0.027
WC (cm)^a^	107.70 ± 8.89	108.09 ± 9.77	106.92 ± 6.87	0.553
Fibro score (kPa)^c^	6.72 (5.00–7.50)	6.00 (5.00–7.37)	6.00 (5.00–8.00)	0.994
CAP score (dB/m)^c^	310.00 (287.00–345.00)	310.00 (285.00–340.00)	324.00 (300.00–350.00)	0.235
FBS (mg/dL)^c^	138.00 (113.00–184.00)	133.00 (113.00–186.25)	151.00 (109.00–177.00)	0.460
Fasting insulin (uIU/mL)^c^	10.90 (8.10–13.80)	10.70 (8.02–14.17)	12.00 (9.50–13.80)	0.396
C-peptide (ng/mL)^c^	1.70 (1.30–2.30)	1.70 (1.30–2.37)	1.90 (1.50–2.30)	0.187
HOMA-IR score^c^	3.84 (2.62–5.19)	3.98 (2.47–5.06)	3.73 (2.97–6.00)	0.396
HbA1c (%)^c^	7.40 (6.70–9.00)	7.30 (6.62–9.00)	8.00 (6.90–9.30)	0.142
AST (U/L)^a^	26.21 ± 10.70	24.92 ± 8.94	28.87 ± 13.41	0.144
ALT (U/L)^c^	20.00 (16.00–27.00)	19.00 (13.50–27.00)	20.00 (17.00–28.00)	0.140
GGT (U/L)^c^	34.00 (27.00–46.00)	32.00 (27.00–42.75)	39.00 (25.00–46.00)	0.347
Total cholesterol (mg/dL)^a^	165.84 ± 36.17	170.20 ± 36.41	156.84 ± 34.52	0.091
HDL-C (mg/dL)^c^	35.00 (29.00–42.00)	35.00 (28.25–41.75)	35.00 (29.00–43.00)	0.665
LDL-C (mg/dL)^c^	88.50 (64.75–114.00)	98.00 (68.00–118.00)	71.00 (61.00–105.00)	0.041
Triglycerides (mg/dL)^c^	167.00 (129.00–249.00)	167.50 (122.50–233.25)	167.00 (133.00–268.00)	0.793
Creatinine (mg/dL)^a^	0.93 ± 0.18	0.95 ± 0.17	0.90 ± 0.20	0.285
TSH (uIU/mL)^c^	1.70 (1.20–2.70)	1.75 (1.10–2.65)	1.70 (1.30–3.30)	0.730
TNF-α (pg/mL)^c^	18.00 (14.10–36.90)	17.75 (13.57–28.47)	18.60 (15.10–51.30)	0.125
Adiponectin (mg/L)^c^	6.60 (4.70–8.70)	6.30 (4.57–8.42)	7.70 (5.50–10.05)	0.081
hs-CRP (mg/L)^c^	2.25 (1.20–4.15)	2.30 (1.20–4.30)	2.20 (1.20–3.70)	0.926
Metformin (%)^b^	88 (92.6)	59 (92.2)	29 (93.5)	0.812
DPP-4 inhibitors (%)^b^	26 (27.4)	10 (15.6)	16 (51.6)	< 0.001
Sulfonylureas or megletinides (%)^b^	36 (37.9)	21 (32.8)	15(48.4)	0.142
Statin (%)^b^	36 (37.9)	23 (35.9)	13 (41.9)	0.572
Use of antihypertensive drugs (%)^b^*	58 (61.1)	37 (57.8)	21 (67.7)	0.352

Data are presented as mean ± SD, median (IQR) or number (%).

*METs* metabolic equivalents, *SBP* systolic blood pressure, *DBP* diastolic blood pressure, *BMI* body mass index, *FFM* fat free mass, *TBW* total body water, *WC* waist circumference, *CAP* controlled attenuation parameter, *FBS* fasting blood sugar, *HOMA-IR* homeostasis model assessment insulin resistance, *HbA1c* hemoglobinA1c, *AST* aspartate aminotransferase, *ALT* alanine aminotransferase, *GGT* gammaglutamyl transferase, *HDL-C* high-density lipoprotein cholesterol, *LDL-C* low-density lipoprotein cholesterol, *TSH* thyroid-stimulating hormone, *TNF-α* tumor necrosis factor alpha, *hs-CRP* high-sensitive C-reactive protein, *DPP-4* dipeptidyl Peptidase 4.

^a^Independent sample t test.

^b^Chi-square test.

^c^Mann–Whitney U test.

*Angiotensin II receptor blockers, beta blockers or angiotensin-converting enzyme inhibitors.

To find the predictive factors that affected hypermetabolism in subjects with both T2DM and NAFLD, parameters with a p-value < 0.2 in univariate analysis (data not shown), including sex, age, physical activity, AST, ALT, cholesterol, TNF-α, adiponectin and FFM, were entered into the multivariate model. According to the results of multivariable logistic regression analysis (Table 2), hypermetabolism was positively associated with adiponectin (odds ratio [OR] 1.17, 95% confidence interval [CI] 1.02–1.34, p = 0.030), physical activity (OR 1.13, 95% CI 1.002–1.28, p = 0.046), ALT (OR 1.06, 95% CI 1.006–1.12, p = 0.031) and diastolic blood pressure (OR 1.07, 95% CI 1.01–1.13, p = 0.021). In contrast, FFM was negatively related to hypermetabolism (OR 0.93, 95% CI 0.88–0.99, p = 0.023).

**Table 2:** Association between demographic, anthropometric and laboratory parameters with hypermetabolism in logistic regression analysis (sample size n = 95).

Parameter	OR	95% CI	p-value
FFM	0.935	0.883–0.991	0.023
Adiponectin	1.167	1.015–1.342	0.030
Physical activity	1.134	1.002–1.284	0.046
ALT (U/L)	1.062	1.006–1.122	0.031
DBP (mmHg)	1.067	1.010–1.127	0.021

*FFM* fat free mass, *ALT* alanine aminotransferase, *DBP* diastolic blood pressure, *OR* odds ratio, *CI* confidence interval.

## Discussion

Although, historically, a negative relationship between obesity and REE has been shown^32^, recent studies have challenged this finding, indicating that obese individuals have higher BMR or REE^33,34^. Likewise, the results of a number of studies conducted on obese participants undergoing bariatric surgery, which caused metabolic and compositional alterations, have shown a significant decrease of BMR^35^. In obese individuals with T2DM, NAFLD, metabolic syndrome and other chronic diseases, increased inflammation and cytokine production, as well as the increased level of reactive oxygen species (ROS) production, play a potential role in increasing BMR, as this process causes mitochondrial disturbance and leads to excess heat generation and a higher level of metabolism^17,36^. For instance, Tarantino et al., showed that morbidly obese NAFLD patients with metabolic syndrome had higher BMR in comparison to NAFLD individuals with a similar weight but without metabolic syndrome^17^. The higher level of BMR in obese NAFLD individuals with metabolic syndrome has been to some extent attributed to low-grade, chronic inflammation^17^.

There is a positive association between diabetes and REE^15,37^. However, there is inadequate evidence regarding the threshold of blood glucose in which REE is increased^38^. Some studies have suggested that the diabetes per se, not the level of glucose level, is a major determinant of high REE in diabetic subjects^37^. Other studies have also indicated that REE is only elevated in uncontrolled diabetic subjects and diabetic cases who took blood glucose lowering drugs experienced a decrease in REE^38–40^. Nevertheless, among subjects with concurrent T2DM and NAFLD, our findings suggested that the effect of other factors, including FFM, adiponectin, diastolic blood pressure, physical activity and ALT, on REE was greater than that of glycemia and insulin levels.

In the present study, an inverse association between FFM and hypermetabolism was shown. Protein turnover is assumed to account for 20% of REE in healthy subjects^41^, and elevated protein turnover is associated with increased REE^42,43^ and loss of FFM^43^. An increase in protein turnover has been previously reported in chronic diseases^44,45^ as well as in diabetes^46^ and liver disease^47^. Therefore, this suggests that the elevated protein turnover, among other factors such as systemic inflammatory response, may be contributing to FFM depletion and increase REE levels in subjects with NALFD and diabetes.

Adiponectin is an adipokine produced by adipose tissue, contributing to the regulation of glucose levels and fatty acid metabolism^48^. The current study suggested that adiponectin was a strong positive determinant for REE in individuals with NAFLD and T2DM. A positive association between adiponectin concentrations and energy expenditure during hyperinsulinemia state among offspring of T2DM individuals^49^, in underweight patients with COPD^50^, and in elderly individuals after resistance exercise^51^ has been shown. However, the literature represents diverse findings in regard to adiponectin levels and REE. One study found that adiponectin concentration had no relationship with energy expenditure among non-diabetic subjects^52^. Another cross sectional study has also reported no relationship between circulating adiponectin and REE in both young and elderly women after adjusting for several confounding factors such as fat mass and FFM^53^. Moreover, some studies have indicated serum adiponectin is negatively correlated with REE in Caucasians^54^ and Pima Indians^55^. To explain these contradictions, the role of genetics as a factor that can influence both energy expenditure and adiponectin^56,57^, the use of different populations in studies, and the different methods used to assess energy expenditure must be taken into account. Based on our literature review, this research, for the first time, addressed the relationship between adiponectin and hypermetabolism among individuals with both T2DM and NAFLD. The mechanisms underlying adiponectin and hypermetabolism are not precisely understood. Some studies suggested that the involvement of adiponectin in energy homeostasis probably mediated through the regulation of uncoupling proteins (UCPs), a family of transporters that present in the mitochondria inner membrane. Animal studies showed that adiponectin administration up-regulates UCP2 expression in the liver tissues of adiponectin knockout mice^58,59^ and this may increase REE^60,61^. In another study, intracerebroventricular administration of adiponectin in mice induced UCP1 mRNA expression and decreasing the body weight, while not affecting the food intake^62^. However, further exploration should be done to discover the role of adiponectin in increasing metabolism and its possible mechanisms.

The results of the present study showed a positive association between diastolic blood pressure and hypermetabolism. In accordance with our findings, Ali et al.^63^ and Snodgrass et al.^64^ showed a direct relationship between BMR and blood pressure. Some possible mechanisms that explain the relationship between BMR and blood pressure are as follows: (1) increased activity of the sympathetic nervous system, which can elevates BMR and blood pressure^64,65^; (2) thyroid hormones levels (T3 and T4) which can affect both blood pressure and metabolism^64,66^; (3) accumulation of reactive oxygen species (ROS) related to the oxidative stress conditions that can elevate BMR and also increase blood pressure through endothelial dysfunction, inflammation and changes in the regulation of nitric oxide^64,67,68^.

In agreement with the previous reports^69,70^, our study indicated a positive association between physical activity and REE. Araiza et al. have shown that REE increases significantly among adults (33 to 69-year-old) with T2DM in response to the 6-week walking program of 10,000 steps on five or more days of the week^70^. There is also evidence showing that resistance training programs increases REE^71–74^.

We found that the higher level of ALT was an independent factor predicting hypermetabolism in T2DM individuals with NAFLD. Although there are limitations in applying ALT in the diagnosis of NAFLD and NASH, it is widely used as a surrogate marker of hepatocellular inflammation and damage in liver diseases^75^, and as it is known, inflammation is significantly related to the elevation of REE^76,77^. While the present study found no association between inflammatory markers (CRP and TNF-α) and hypermetabolism, the effect of inflammatory markers cannot be completely ruled out; this is because, in this study, we did not examine all inflammatory markers such as IL-6, etc. In addition, all of these inflammatory markers are checked in the serum of subjects, and it does not necessarily show the inflammatory microenvironment of the liver.

To the best of our knowledge, this is the first study which evaluated hypermetabolism and its contributing factors in T2DM individuals with NAFLD. However, our study has some limitations. The cross-sectional design of the current study made it impossible to draw a conclusion on causality. Furthermore, our small sample size might have affected the results. We should also consider the limitations of the device used for measuring body composition. For instance, bioelectrical impedance can be affected by some factors including the level of body hydration, body geometry, etc., which could result in the incorrect estimation of body composition^78^.

## Conclusion

In patients with NAFLD and T2DM, hypermetabolism has a direct association with adiponectin, physical activity, ALT and diastolic blood pressure, but a diverse relationship with FFM.

## Data Availability

The datasets used and/or analysed during the current study available from the corresponding author on reasonable request.

## References

[1] García-Monzón C (2015). Prevalence and risk factors for biopsy-proven non-alcoholic fatty liver disease and non-alcoholic steatohepatitis in a prospective cohort of adult patients with gallstones. Liver Int..

[2] Zheng Y, Ley SH, Hu FB (2018). Global aetiology and epidemiology of type 2 diabetes mellitus and its complications. Nat. Rev. Endocrinol..

[3] Kotronen A (2008). Liver fat is increased in type 2 diabetic patients and underestimated by serum alanine aminotransferase compared with equally obese nondiabetic subjects. Diabetes Care.

[4] Bica C (2020). Non-alcoholic fatty liver disease: A major challenge in type 2 diabetes mellitus. Exp. Ther. Med..

[5] Tanase DM (2020). The intricate relationship between type 2 diabetes mellitus (T2DM), insulin resistance (IR), and nonalcoholic fatty liver disease (NAFLD). J. Diabetes Res..

[6] Younossi ZM (2016). Global epidemiology of nonalcoholic fatty liver disease—Meta-analytic assessment of prevalence, incidence, and outcomes. Hepatology.

[7] Ciardullo S, Perseghin G (2021). Statin use is associated with lower prevalence of advanced liver fibrosis in patients with type 2 diabetes. Metabolism.

[8] Yabiku K (2021). Efficacy of sodium-glucose cotransporter 2 inhibitors in patients with concurrent type 2 diabetes mellitus and non-alcoholic steatohepatitis: A review of the evidence. Front. Endocrinol. (Lausanne).

[9] Lamos EM (2021). Effects of anti-diabetic treatments in type 2 diabetes and fatty liver disease. Expert Rev. Clin. Pharmacol..

[10] Mertens J (2021). NAFLD in type 1 diabetes: Overrated or underappreciated?. Therap. Adv. Endocrinol. Metab..

[11] Arrese M, Barrera F (2019). Concurrent nonalcoholic fatty liver disease and type 2 diabetes: Diagnostic and therapeutic considerations. Expert Rev. Gastroenterol. Hepatol..

[12] Bril F, Cusi K (2017). Management of nonalcoholic fatty liver disease in patients with type 2 diabetes: A call to action. Diabetes Care.

[13] Miele L (2016). Nonalcoholic fatty liver disease as trigger of cardiovascular and metabolic complication in metabolic syndrome. Intern. Emerg. Med..

[14] Sampath Kumar A (2019). Correlation between basal metabolic rate, visceral fat and insulin resistance among type 2 diabetes mellitus with peripheral neuropathy. Diabetes Metab. Syndr..

[15] Huang KC (2004). Resting metabolic rate in severely obese diabetic and nondiabetic subjects. Obes. Res..

[16] Reddavide R (2019). Non-alcoholic fatty liver disease is associated with higher metabolic expenditure in overweight and obese subjects: A case-control study. Nutrients.

[17] Tarantino G (2008). Basal metabolic rate in morbidly obese patients with non-alcoholic fatty liver disease. Clin. Investig. Med..

[18] Herrera-Amante CA (2021). Development of alternatives to estimate resting metabolic rate from anthropometric variables in paralympic swimmers. J. Sports Sci..

[19] Sabounchi NS, Rahmandad H, Ammerman A (2013). Best-fitting prediction equations for basal metabolic rate: Informing obesity interventions in diverse populations. Int. J. Obes..

[20] Hipskind P (2011). Do handheld calorimeters have a role in assessment of nutrition needs in hospitalized patients? A systematic review of literature. Nutr. Clin. Pract..

[21] Bitz C (2004). Increased 24-h energy expenditure in type 2 diabetes. Diabetes Care.

[22] Fontvieille A (1992). Twenty-four-hour energy expenditure in Pima Indians with type 2 (non-insulin-dependent) diabetes mellitus. Diabetologia.

[23] Piaggi P (2015). Fasting hyperglycemia predicts lower rates of weight gain by increased energy expenditure and fat oxidation rate. J. Clin. Endocrinol. Metab..

[24] Huang Z (2021). Validation of controlled attenuation parameter measured by fibroscan as a novel surrogate marker for the evaluation of metabolic derangement. Front. Endocrinol..

[25] American Diabetes Association (2017). Classification and diagnosis of diabetes: Standards of medical care in diabetes. Diabetes Care.

[26] Patel PJ (2017). Alcohol consumption in diabetic patients with nonalcoholic fatty liver disease. Can. J. Gastroenterol. Hepatol..

[27] Lombardo M (2021). Changes in eating habits and physical activity after COVID-19 pandemic lockdowns in Italy. Nutrients.

[28] Lee PH (2011). Validity of the international physical activity questionnaire short form (IPAQ-SF): A systematic review. Int. J. Behav. Nutr. Phys. Act..

[29] Delsoglio M (2019). Indirect calorimetry in clinical practice. J. Clin. Med..

[30] Harris JA, Benedict FG (1918). A biometric study of human basal metabolism. Proc. Natl. Acad. Sci..

[31] Jouinot A (2018). Resting energy expenditure in the risk assessment of anticancer treatments. Clin. Nutr..

[32] Ravussin E (1988). Reduced rate of energy expenditure as a risk factor for body-weight gain. N. Engl. J. Med..

[33] Carneiro IP (2016). Is obesity associated with altered energy expenditure?. Adv. Nutr..

[34] Tarantino G, Savastano S, Colao A (2010). Hepatic steatosis, low-grade chronic inflammation and hormone/growth factor/adipokine imbalance. World J. Gastroenterol..

[35] Sheikhi A (2020). Effect of bariatric surgeries on metabolic rate, a systematic review and meta-analyses. Ann. Bariatr. Surg..

[36] Esser N (2014). Inflammation as a link between obesity, metabolic syndrome and type 2 diabetes. Diabetes Res. Clin. Pract..

[37] Martin K (2004). Estimation of resting energy expenditure considering effects of race and diabetes status. Diabetes Care.

[38] Ryan M (2006). Resting energy expenditure is not increased in mildly hyperglycaemic obese diabetic patients. Br. J. Nutr..

[39] Franssila-Kallunki A, Groop L (1992). Factors associated with basal metabolic rate in patients with type 2 (non-insulin-dependent) diabetes mellitus. Diabetologia.

[40] Gougeon R (2002). The prediction of resting energy expenditure in type 2 diabetes mellitus is improved by factoring for glycemia. Int. J. Obes..

[41] Welle S, Nair K (1990). Relationship of resting metabolic rate to body composition and protein turnover. Am. J. Physiol.-Endocrinol. Metab..

[42] Berclaz P-Y (1996). Changes in protein turnover and resting energy expenditure after treatment of malaria in Gambian children. Pediatr. Res..

[43] Kao CC (2011). Resting energy expenditure and protein turnover are increased in patients with severe chronic obstructive pulmonary disease. Metabolism.

[44] Engelen MP (2000). Enhanced levels of whole-body protein turnover in patients with chronic obstructive pulmonary disease. Am. J. Respir. Crit. Care Med..

[45] Biolo G (2003). Mechanisms of altered protein turnover in chronic diseases: A review of human kinetic studies. Curr. Opin. Clin. Nutr. Metab. Care.

[46] Gougeon R (2008). Determinants of whole-body protein metabolism in subjects with and without type 2 diabetes. Diabetes Care.

[47] Greer R (2003). Body composition and components of energy expenditure in children with end-stage liver disease. J. Pediatr. Gastroenterol. Nutr..

[48] Yanai H, Yoshida H (2019). Beneficical effect of adiponectin on glucose and lipid metabolism and atherosclerothic progression: Mechanisms and perspectives. Int. J. Mol. Sci..

[49] Salmenniemi U (2005). Association of adiponectin level and variants in the adiponectin gene with glucose metabolism, energy expenditure, and cytokines in offspring of type 2 diabetic patients. J. Clin. Endocrinol. Metab..

[50] Brúsik M (2012). Circulatory and adipose tissue leptin and adiponectin in relationship to resting energy expenditure in patients with chronic obstructive pulmonary disease. Physiol. Res..

[51] Fatouros IG (2009). Intensity of resistance exercise determines adipokine and resting energy expenditure responses in overweight elderly individuals. Diabetes Care.

[52] Stefan N (2002). Plasma adiponectin levels are not associated with fat oxidation in humans. Obes. Res..

[53] Usui C (2007). Relationship between blood adipocytokines and resting energy expenditure in young and elderly women. J. Nutr. Sci. Vitaminol..

[54] Ruige JB (2005). Resting metabolic rate is an important predictor of serum adiponectin concentrations: Potential implications for obesity-related disorders. Am. J. Clin. Nutr..

[55] Pannacciulli N (2006). Lower total fasting plasma adiponectin concentrations are associated with higher metabolic rates. J. Clin. Endocrinol. Metab..

[56] Li G, Zhong L (2022). Genetic variations in adiponectin levels and dietary patterns on metabolic health among children with normal weight versus obesity: The BCAMS study. Int. J. Obes..

[57] Loos RJ (2007). Adiponectin and adiponectin receptor gene variants in relation to resting metabolic rate, respiratory quotient, and adiposity-related phenotypes in the Quebec Family Study. Am. J. Clin. Nutr..

[58] Finelli C, Tarantino G (2013). What is the role of adiponectin in obesity related non-alcoholic fatty liver disease?. World J. Gastroenterol..

[59] Zhou M (2008). Mitochondrial dysfunction contributes to the increased vulnerabilities of adiponectin knockout mice to liver injury. Hepatology.

[60] Taghadomi Masoumi Z (2018). Association between uncoupling protein 2, adiponectin and resting energy expenditure in obese women with normal and low resting energy expenditure. Gynecol. Endocrinol..

[61] Moradi S (2021). The effect of omega3 fatty acid supplementation on PPARγ and UCP2 expressions, resting energy expenditure, and appetite in athletes. BMC Sports Sci. Med. Rehabil..

[62] Qi Y (2004). Adiponectin acts in the brain to decrease body weight. Nat. Med..

[63] Ali N (2017). Hypertension prevalence and influence of basal metabolic rate on blood pressure among adult students in Bangladesh. BMC Public Health.

[64] Snodgrass JJ (2008). The influence of basal metabolic rate on blood pressure among indigenous Siberians. Am. J. Phys. Anthropol..

[65] Luke A (2004). Association between blood pressure and resting energy expenditure independent of body size. Hypertension.

[66] Danzi S, Klein I (2003). Thyroid hormone and blood pressure regulation. Curr. Hypertens. Rep..

[67] Rathaus M, Bernheim J (2002). Oxygen species in the microvascular environment: Regulation of vascular tone and the development of hypertension. Nephrol. Dial. Transplant.

[68] Rodríguez-Iturbe B (2004). Oxidative stress, renal infiltration of immune cells, and salt-sensitive hypertension: All for one and one for all. Am. J. Physiol. Renal Physiol..

[69] Starling RD (2001). Energy expenditure and aging: Effects of physical activity. Int. J. Sport Nutr. Exerc. Metab..

[70] Araiza P (2006). Efficacy of a pedometer-based physical activity program on parameters of diabetes control in type 2 diabetes mellitus. Metabolism.

[71] Hunter GR (2000). Resistance training increases total energy expenditure and free-living physical activity in older adults. J. Appl. Physiol..

[72] Melby C (1993). Effect of acute resistance exercise on postexercise energy expenditure and resting metabolic rate. J. Appl. Physiol..

[73] Pratley R (1994). Strength training increases resting metabolic rate and norepinephrine levels in healthy 50-to 65-yr-old men. J. Appl. Physiol..

[74] Treuth MS (1995). Energy expenditure and substrate utilization in older women after strength training: 24-h calorimeter results. J. Appl. Physiol..

[75] Ma X (2020). Proportion of NAFLD patients with normal ALT value in overall NAFLD patients: A systematic review and meta-analysis. BMC Gastroenterol..

[76] Utaka S (2005). Inflammation is associated with increased energy expenditure in patients with chronic kidney disease. Am. J. Clin. Nutr..

[77] Agarwal R (2005). Smoking, oxidative stress and inflammation: Impact on resting energy expenditure in diabetic nephropathy. BMC Nephrol..

[78] Deurenberg P (1996). Limitations of the bioelectrical impedance method for the assessment of body fat in severe obesity. Am. J. Clin. Nutr..

